# Explainable AI–based prognostication of patients with resectable colorectal liver metastases using preoperative clinical parameters: is it comparable to traditional clinical scoring systems?

**DOI:** 10.3389/fonc.2026.1890766

**Published:** 2026-09-21

**Authors:** Nikhil Pramod Kumar, Sarkis Drejian, Mehdi Sadat Akhavi, Hemin Ali Qadir, Åsmund Avdem Fretland, Airazat M. Kazaryan, Davit Aghayan, Ilangko Balasingham, Bjørn Edwin

**Affiliations:** 1 The Intervention Centre, Oslo University Hospital, Oslo, Norway; 2 Institute of Clinical Medicine, University of Oslo, Oslo, Norway; 3 Department of HPB Surgery, Oslo University Hospital, Oslo, Norway; 4 Department of Surgery, Østfold Hospital Trust, Grålum, Norway; 5 Department of Abdominal Surgery, Yerevan State Medical University After M. Heratsi, Yerevan, Armenia; 6 Department of Faculty Surgery N2, I. M. Sechenov First Moscow State Medical University, Moscow, Russia; 7 Department of Surgery, Vestre Viken Hospital Trust, Ringerike Hospital, Hønefoss, Norway; 8 Department of Electronic Systems, Norwegian University of Science and Technology, Trondheim, Norway

**Keywords:** colorectal liver metastases, deep learning, machine learning, model interpretability, survival prediction

## Abstract

**Background:**

Prognostication for patients undergoing liver resection for colorectal liver metastases (CRLM) remains challenging. Artificial intelligence–based survival models may improve individualized risk estimation.

**Methods:**

We developed and compared classical machine learning and deep learning survival models using exclusively preoperative clinical variables from 350 patients undergoing first-time liver resection for CRLM. Models included Cox proportional hazards (CoxPH), random survival forest (RSF), support vector machine for survival (SVM), XGBoost survival, DeepSurv, and DeepHit. Model performance was evaluated using the concordance index (C-index) and the Integrated Brier Score (IBS) on a training set of 245 patients, testing set of 105 patients, and an independent internal validation set of 88 patients from the OSLO-COMET trial. Model interpretability was assessed using SHapley Additive explanation (SHAP) analysis. The results of the developed models were compared to well established Basingstoke Predictive Index (BPI).

**Results:**

On internal validation cohort, CoxPH demonstrated the most consistent performance on the internal validation set (C-index 0.59), and on the test set patients (C-index 0.65), comparable to more complex machine learning and deep learning models. Although RSF achieved the highest training performance, this did not translate into superior performance both on the test and the internal validation datasets. CoxPH and DeepSurv models showed comparable 1-, 3-, and 5-year individual prediction to BPI. SHAP analysis consistently identified ASA score, lobar distribution, primary tumor location and number of metastases as the most influential predictors across models.

**Conclusions:**

Using structured preoperative clinical data, we developed machine-and deep-learning models to predict overall survival in patients with CRLM. Given that the models were based exclusively on preoperative clinical variables, their performance is encouraging. Predictive accuracy may be further improved by incorporating additional data modalities, particularly radiological features as well as by training on larger datasets.

## Introduction

Colorectal cancer represents the third most commonly diagnosed malignancy worldwide. Approximately half of all patients with colorectal cancer will, at some point during their disease course, develop liver metastases, accounting for nearly two-thirds of all colorectal cancer-related deaths (1).

Curative-intent treatment modalities for colorectal liver metastases (CRLM) include liver resection (LR), which is the gold standard. Other treatments methods are thermal ablation and systemic chemotherapy/immunotherapy, either alone or in combination with surgery. Liver transplantation is an option in case of non-resectable CRLMs (2–5). The optimal therapeutic strategy relies on a comprehensive evaluation of a wide range of clinical, histopathological, and molecular features to enable a tailored, patient-specific approach (6). However, in routine clinical setup, not all the aforementioned features could be available for the evaluation of the patients with CRLM, therefore relying on clinical parameters can be more practical.

Prior to the introduction of the artificial intelligence (AI), several conventional predictive models have been developed and used to estimate prognosis and guide treatment in CRLM. Among the most widely adopted are the Fong Clinical Risk Score and the Basingstoke Predictive Index (BPI) (7, 8), which integrate preoperative and postoperative clinical and histopathological variables, essential in predicting recurrence and overall survival. However, over the past three decades, AI has increasingly gained an important role as a prognostic tool in the healthcare system. The strength of AI lies in detecting complex, non-linear interactions between variables more efficiently than conventional methods. AI has shown promise not only in prognostication but also across diagnostics, treatment planning and clinical decision support (9, 10), driving ongoing efforts to develop novel AI-based predictive models.

Majority of recent studies, including those published by Lam et al. and Ding et al. showed promising results by developing machine-learning models for post-hepatectomy prognosis in CRLM (11, 12). However, these models typically incorporate preoperative, perioperative, and postoperative variables. In contrast, our aim was to develop both classical machine-learning and deep-learning models based exclusively on preoperative parameters. This approach reflects real-world clinical practice, where only preoperative information is available during multidisciplinary team (MDT) discussions. Accordingly, our models were intentionally restricted from incorporating perioperative and postoperative data.

The objective of this study was to develop and evaluate machine-learning and deep-learning based survival models for patients with CRLM using exclusively preoperative clinical variables, with the aim of providing a complementary tool for MDT decision-making. In addition, we sought to estimate individualized 1-, 3-, and 5-year survival probabilities and to compare model performance with the well-established traditional Basingstoke Predictive Index (BPI). Such models may provide valuable support for multidisciplinary discussions and enhance clinical decision-making.

## Methods

### Data collection

A total of 350 patients, who underwent first-time liver resection for CRLM between January 2016 and December 2020, were identified from a prospectively maintained database and included in this study (**Figure 1**). All surgical procedures were performed laparoscopically at Rikshospitalet, Oslo University Hospital, a tertiary referral center for hepatopancreatobiliary surgery. Patients operated with a liver-first approach (metastatic tumor liver resection before primary tumor resection) were excluded from the analysis. The study was approved by the Institutional Review Board, and due to its retrospective design, the requirement for written informed consent was waived.

### Data preprocessing

The dataset was preprocessed to ensure consistency, analytical validity, and clinical relevance of the variables included in the survival analysis. The selected variables are presented in **Table 1**.

**Table 1:** Baseline characteristics for the studied cohort and the internal validation (OSLO-COMET) cohort.

Variables	*n* = 350	*n* = 88
Number of events (death)	158	53
Median age (years)	68.5	70
Gender (male)	213 (61%)	35 (40%)
Median CEA level (IQR), µg/L	25.4 (22.8 – 28.02)	3.6 (2.4 – 9)
Synchronous	240 (69%) *1 missing	53 (60.2%)
ASA		
- 1	4 (1%)	7 (8%)
- 2	191 (55%)	36 (41%)
- 3	151 (43%)	37 (42%)
- 4	2 (0.5%) *2 missing	1 (1.1%) *7 missing
Median tumor size (IQR), CM	2.25 (1.4 to 3.1)	2.5 (1.5 to 3.6)
Minimum (maximum)	1 (17) *4 missing	0.6 (7)
Multiple metastases	129 (39%)	35 (40%)
Maximum	10 * 3 missing	7 * 2 missing
Primary tumor AJCC stage		
- T1	7 (2%)	2 (2.3%)
T2	37 (11%)	5 (5.7%)
- T3	166 (47%)	54 (61.4%)
- T4	73 (21%) * 68 missing	17 (19.3%) * 10 missing
Lobar distribution		
- Right	170 (49%)	46 (52.3%)
- Left	107 (31%)	23 (26.1%)
- Both	71 (20%) * 2 missing	19 (21.6%)
Primary tumor location		
- Right	84 (24%)	19 (21.6%)
- Left	130 (37%)	32 (36.4%)
- Rectum	110 (31%)	30 (34.1%)
- Transversum	10 (3%)	4 (4.5%)
- Multiple	14 (4%) * 2 missing	0 * 3 missing
Primary tumor lymph node	197 (56%) * 28 missing	54 (61.4%) * 10 missing
Dukes		
- A	8 (2%)	1 (1.1%)
- B	62 (18%)	19 (21.6%)
- C	87 (25%)	46 (52.3%)
- D	179 (42%) * 14 missing	8 (9.1%) *14 missing
Previous abdominal surgery (except liver)	342 (98%)	
Extrahepatic metastases	57 (16%)	10 (11.4%)
Neoadjuvant chemotherapy for liver	147 (42%) * 1 missing	30 (34.1%)

* = 1 missing value for each variable.

Categorical variables were converted into two groups based on standard clinical classifications. The American Society of Anesthesiologists (ASA) score was grouped into low risk (ASA 1–2) and high risk (ASA 3–4) to reflect overall patient health. These groupings were also chosen because some original categories had very few patients, which could make the survival analysis unreliable.

Carcinoembryonic antigen (CEA) level was divided into two groups using a clinical threshold of 60 µg/L as reported previously (13). The number of liver metastases was grouped as 1, 2, or ≥ 3 to reflect increasing disease burden in a clinically meaningful way. This preprocessing approach enabled the retention of clinically interpretable features while ensuring the dataset was suitable for downstream survival modeling.

### Feature selection (univariate Cox proportional hazard)

To identify relevant predictors for survival analysis, we performed univariate Cox Proportional Hazard (CoxPH) on the training dataset (14). Variables with p-values greater than 0.05 were excluded from further consideration, thereby retaining only those features that demonstrated a potential association with survival outcomes as listed in **Table 2**.

**Table 2:** Univariate analysis for overall survival on the training set.

Variable	n_used 245	Hazard ratio (HR)	Lower 95% CI	Upper 95% CI	P-value	Global P-value
Age	245	1.0445	1.0236	1.0658	0.0002	–
Maximum tumor size	242 * 3 missing	1.0118	1.0034	1.0204	0.0058	–
ASA	243 * 2 missing	2.1584	1.4719	3.1652	<0.0001	–
CEA	230 *15 missing	2.5809	1.3398	4.9713	0.0046	–
T-status	199 * 146 missing	0.9378	0.5201	1.6907	0.8307	–
Gender	245	0.8333	0.5655	1.2278	0.3563	–
Previous laparoscopic surgery	244 * 1 missing	0.8656	0.5923	1.2650	0.4560	–
Previous open surgery	245	1.0640	0.7295	1.5515	0.7478	–
Extrahepatic metastasis	245	1.9246	1.2463	2.9721	0.0031	–
Lymph node involvement in the primary tumor	227 * 18 missing	1.5209	1.0163	2.2761	0.0415	–
Liver metastasis diagnosis (synchronous)	244 * 1 missing	1.4307	0.9403	2.1766	0.0944	–
Previous abdominal surgery	245	0.6421	0.2036	2.0245	0.4496	–
Neoadjuvant chemotherapy	244 * 1 missing	1.3045	0.8971	1.8968	0.1640	–
Number of metastases_1	244	ref	ref	ref	ref	0.0468
Number of metastases_2	* 1 missing	1.7503	1.1389	2.6898	0.0107	
Number of metastases_3		1.2205	0.7201	2.0686	0.4593	
*Repeated laparoscopy_0	245	ref	ref	ref	ref	0.6863
Repeated laparoscopy_1		1.2762	0.7152	2.2770	0.4091	
Repeated laparoscopy_2		0.7454	0.1038	5.3507	0.7701	
Dukes						
A	236	ref	ref	ref	ref	0.1594
B	* 9 missing	0.8672	0.2006	3.7500	0.8488	
C		1.3821	0.3270	5.8422	0.6599	
D		1.5365	0.3742	6.3075	0.5511	
PTL						
Right colon	244	ref	ref	ref	ref	0.0320
Left colon	* 1 missing	0.4593	0.2839	0.7431	0.0015	
Rectum		0.7249	0.4578	1.1477	0.1700	
Transversum		0.5216	0.1604	1.6966	0.2795	
Multiple		0.5799	0.1783	1.8853	0.3650	
Lobar distribution	244					0.0142
Right	* 1 missing	ref	ref	ref	ref	
Left		1.7832	1.1609	2.7391	0.0083	
Both		1.7075	1.0508	2.7747	0.0308	

ASA, American Society of Anesthesiologists; CEA, Carcinoembryonic Antigen; PTL, Primary Tumor Location.

*Repeated laparoscopic surgeries before first liver resection.

To ensure the integrity of the analysis and assess the robustness of our findings, we performed a sensitivity analysis following data imputation. The analysis demonstrated that the same variables remained statistically significant after imputation, indicating that the results were consistent and robust despite the handling of the missing data, as presented in **Supplementary Table 1**.

Based on the results of the univariate CoxPH analysis, the variables (**Table 2**) that met the significance threshold and had p ≤ 0.05 were retained for downstream modeling. The Cox proportional hazards analyses were conducted using the Lifelines Python library (15).

### Imputation

To address missing values, we applied multivariate imputation by chained equations (MICE) using a machine learning-based approach (16). Categorical variables the ASA score, CEA level, primary tumor location (PTL) and lymph node involvement were imputed using a random forest classifier (17). Continuous variables, including age and maximum tumor size, were imputed using Bayesian ridge regression (18, 19), implemented through the scikit-learn library in Python (20). This approach was chosen to preserve the underlying structure and variability of the data while avoiding the biases often introduced by simpler imputation methods. To prevent information leakage, the imputation model was fit exclusively on the training data, and the learned imputation parameters were subsequently applied using transform only to the test set and the internal validation OSLO-COMET dataset, ensuring that no information from either set influenced the imputation model.

### Modeling and evaluation

To assess the prognostic value of selected clinical features, we developed and compared a range of statistical, machine-learning and deep-learning models for survival analysis; these models were trained to predict time-to-event outcomes using our processed dataset.

#### Machine learning models

We used four machine-learning models from the scikit-survival library (version 0.12) (21), which offers reliable and censoring aware implementations suited for time-to-event analysis.

Cox Proportional Hazards Model (CoxPH) is a semi-parametric model that relates patient features to the hazard of experiencing an event. It is widely used, easy to interpret, and serves as a natural statistical baseline for comparing more flexible models. For this study, it was implemented alongside the machine-learning models to allow consistent training and evaluation across all approaches (14).

Random Survival Forest (RSF) is an ensemble of survival trees that repeatedly splits the data to uncover meaningful patterns. By averaging predictions across many trees, RSF can capture complex, non-linear relationships between variables without assuming proportional hazards (22).

Support Vector Machine for Survival (SVM) adapts the support vector machine framework to handle censored outcomes. It uses a survival-specific pairwise ranking hinge loss, which compares individuals whose event ordering is known, enabling the model to incorporate censored observations while learning non-linear risk boundaries (23).

XGBoost for Survival applies gradient boosting to survival data using a cox partial-likelihood loss. By building trees sequentially and correcting errors at each step, it learns flexible and additive patterns that often improve performance on structured clinical datasets (24–26).

Together, these models represent a mix of classical and modern machine-learning strategies, offering a broad view of how different algorithms handle censored survival data.

#### Deep learning models

We implemented two neural network-based survival models using the PyCox framework (27), which provides architectures and loss functions designed specifically for censored data.

DeepSurv is a fully connected neural network that extends the traditional Cox model. Instead of assuming a simple linear relationship between features and the hazard, it learns non-linear transformations that can better capture complex clinical patterns. The model is trained using the same negative partial log-likelihood as CoxPH, allowing it to estimate individualized risk scores flexibly (28).

DeepHit directly models the probability of an event occurring at each discrete time point. To do this, it uses a multi-task loss that combines two components:

1. A likelihood term that helps the model learn the survival probability across time.
2. A ranking term that teaches it to order patients correctly based on risk.

By combining these objectives, DeepHit can learn both absolute survival probabilities and relative risk relationships, making it a powerful option when the underlying hazard is complex or non-proportional (29).

### Hyperparameter optimization

Model hyperparameters were optimized using the Optuna framework (30), which applies Bayesian optimization to efficiently explore parameter space. The dataset was partitioned into a training set (70%) and a test set (30%). Hyperparameter tuning and cross-validation were performed exclusively on the training set to prevent information leakage and reduce overfitting. Final performance was evaluated both on the test set and on an independent internal validation dataset. The internal validation set is 88 laparoscopically operated patients from the OSLO-COMET trial (**Figure 1**). OSLO-COMET (ClinicalTrials.gov: NCT01516710) was an investigator-initiated, open-label, single-center, randomized controlled trial, conducted between 2012 and 2016, that recruited patients from Oslo University Hospital in Oslo, Norway, and does not contain any overlapping patients with the training and test datasets. It was approved by the Regional Ethical Committee of South Eastern Norway (REK Sør-Øst B 2011/1285) and the data protection officer of the Oslo University Hospital. The baseline characteristics of OSLO-COMET patients are presented in **Table 1** (31). For reproducibility, the optimized hyperparameter search space and hyperparameter settings for all models are presented in **Supplementary Table 7**.

**Figure 1:**
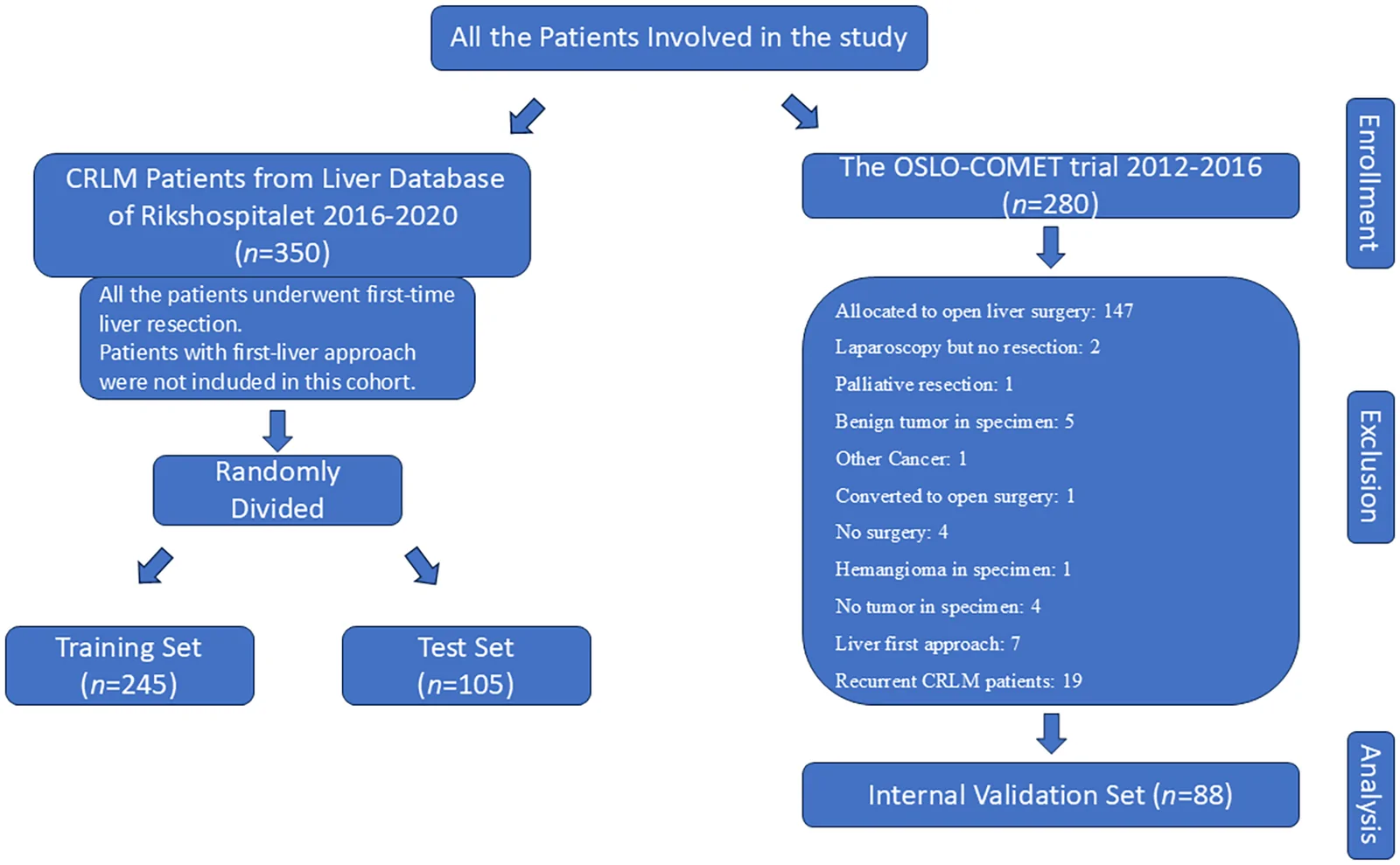
Patient selection flowchart.

Descriptive Kaplan–Meier survival curves were generated to summarize overall survival in the studied cohort and the independent internal validation cohort. Survival time was defined as the interval from diagnosis of CRLM to death or last follow-up, with patients censored at the last known follow-up. The corresponding Kaplan–Meier curves are shown in **Figure 2**.

**Figure 2:**
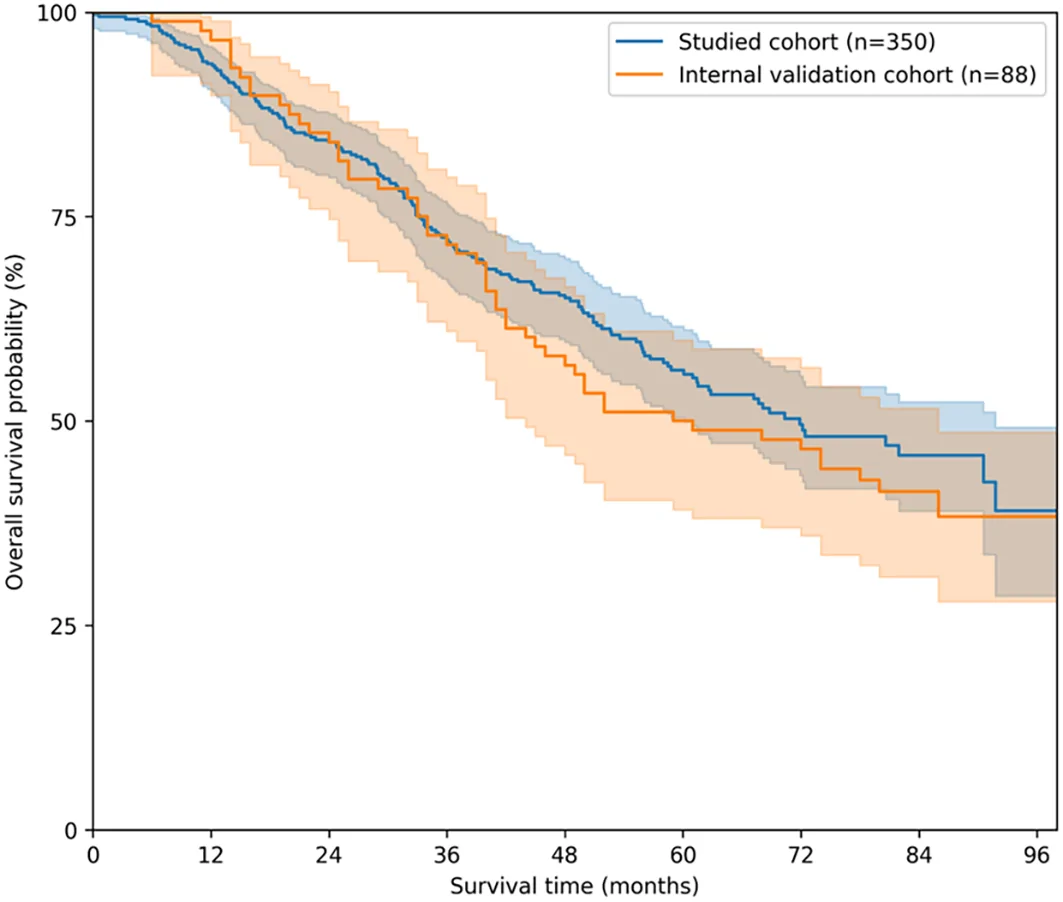
Kaplan-Meier survival curves. Studied cohort: Median overall survival: 72.18 months (95% CI: 91.76 to 61.40). Median follow-up: 68.34 months (95% CI: 70.60 to 62.39). Internal validation cohort: Median overall survival: 59.00 months (95% CI: 86.00 to 44.00). Median follow-up: 92.00 months (95% CI: 99.00 to 86.00). Log-rank test: p-value: 0.4183. Patients at risk, *n*, Studied cohort (350, 328, 283, 241, 191, 122,72, 35, 6). Internal validation cohort (88, 86, 76, 64, 51, 44, 42, 28, 13).

### Evaluation metric

Model performance was primarily evaluated using the C-index (32), and the Integrated Brier Score (IBS) (33), two most widely accepted evaluation measures for survival models in clinical research. The C-index quantifies the proportion of all comparable patient pairs for which the model correctly ranks their survival times. A value of 0.5 indicates random prediction, while 1.0 indicates perfect discrimination. The IBS measures the accuracy of predicted survival probabilities over the entire follow-up period by integrating the Brier score across time, serving as a measure of overall probabilistic prediction error. An IBS value of 0 indicates perfect prediction, with lower values reflecting better predictive performance.

Mean and standard deviation of the C-index and IBS from cross-validation on the training set are presented in **Table 3**. For the test set and the internal validation set, where only a single evaluation is possible, 95% confidence intervals were estimated via bootstrapping (n = 1000 resamples).

**Table 3:** Comparison of model performance based on C-index and IBS.

Model	Training set 245		Test set 105		Internal validation OSLO-COMET 88	
	C index mean ± std	IBS mean ± std	C index mean (95% CI)	IBS mean (95% CI)	C index mean (95% CI)	IBS mean (95% CI)
CoxPH	0.66641 ± 0.04502	0.17677 ± 0.02233	0.65177 (0.57047, 0.73063)	0.16642 (0.14099, 0.19320)	0.59331 (0.50029, 0.67938)	0.42638 (0.32050, 0.54897)
XGBoost	0.64967 ± 0.04198	0.20014 ± 0.04465	0.59887 (0.51692, 0.68326)	0.19613 (0.15866, 0.23947)	0.56341 (0.47802, 0.64710)	0.46180 (0.34995, 0.58887)
SVM	0.67590 ± 0.03248	0.17778 ± 0.03349	0.60441 (0.52542, 0.67906)	0.19022 (0.15667, 0.22478)	0.58989 (0.51077, 0.66999)	0.38628 (0.31066, 0.49398)
RSF	0.68360 ± 0.03698	0.18376 ± 0.03485	0.63544 (0.55803, 0.71331)	0.18573 (0.15460, 0.22094)	0.57709 (0.48966, 0.66519)	0.39550 (0.31444, 0.48709)
DeepHit	0.61415 ± 0.18717	0.20500 ± 0.02739	0.59198 (0.51526, 0.66390)	0.21240 (0.19057, 0.23628)	0.58835 (0.50139, 0.66995)	0.23688 (0.20744, 0.26756)
DeepSurv	0.67120 ± 0.11565	0.15857 ± 0.03641	0.63092 (0.54995, 0.71044)	0.17373 (0.15268, 0.19614)	0.57992 (0.49208, 0.66702)	0.20431 (0.17757, 0.23358)

### Statistical comparison between the models

Pairwise statistical comparisons among the six survival models, including CoxPH, RSF, SVM, XGBoost, DeepSurv, and DeepHit, were performed using paired bootstrap resampling. For each evaluation cohort, including the test and the internal validation sets, and each of the 15 possible model pairs, 1, 000 bootstrap resamples of patients were generated. Within each resample, both models were evaluated on the same patients, thereby accounting for the paired nature of the comparisons and enabling estimation of the distribution of model-to-model differences. Model performance was assessed using two metrics: C-index for discriminative ability and a three-point Integrated Brier Score from predictions at 12, 36, and 60 months as a measure of lower prediction error. Two-sided p-values were derived from the bootstrap distribution of paired differences and adjusted for multiple comparisons using the Holm Bonferroni method (34) across the 60 comparisons (2 cohorts × 15 model pairs × 2 metrics).

### Model interpretability

To interpret the contribution of individual predictors, SHapley Additive exPlanations (SHAP) was implemented (35). SHAP assigns an importance value to each feature based on its marginal contribution to the predicted risk for each patient. The explainer type, prediction output being explained, and background reference dataset were selected according to each model’s architecture and are reported for each model in **Supplementary Table 6**. SHAP explanations were generated independently for the test and the internal validation sets. This approach enhances the interpretability of complex models by identifying the predictors contributing most strongly to individual risk predictions and thereby facilitates the identification of clinically meaningful predictors. Such model interpretability has potential for supporting clinical interpretation.

### Testing of CoxPH, DeepSurv and RSF models, and comparison with BPI score

We individually predicted 1-, 3-, and 5- survival probabilities for the 245-training set, the 105-test set and the 88-internal validation OSLO-COMET patients. Survival predictions were generated using the CoxPH, DeepSurv and RSF models. We also individually predicted 1-, 3-, and 5-year survival probabilities for all the three sets of patients using BPI score. The comparison of the results between BPI score and CoxPH, DeepSurv and RSF models is presented in **Supplementary Tables 3**–**S5**, for illustrative purposes.

## Results

We evaluated the predictive performance of CoxPH model alongside several machine-learning and deep-learning–based survival models for predicting overall survival. The analysis included 350 patients for model building with a training set of 245 patients and a test set of 105 patients, used to assess generalizability, and 88 patients from the OSLO-COMET dataset reserved for internal validation. The median OS for the 350 patients was 72 months, and for the internal validation OSLO-COMET cohort was 59 months. Model performance, measured using the C-index and IBS, is summarized in **Table 3**.

On the training set, the RSF model achieved the highest mean C-index (0.6836 ± 0.0370), followed by SVM (0.6759 ± 0.0325), and DeepSurv (0.6712 ± 0.1157), while CoxPH model demonstrated a comparable C-index (0.6664 ± 0.045). In terms of prediction error, DeepSurv achieved the lowest IBS on the training set (0.1586 ± 0.0364), followed by CoxPH (0.1768 ± 0.0223) and SVM (0.1778 ± 0.0335).

On the test set, CoxPH achieved the highest C-index (0.6518, 95% CI: 0, 5705-0.7306), followed by RSF (0.6354, 95% CI: 0.5580-0.7133) and DeepSurv (0.6309, 95% CI: 0.5500-0.7104). CoxPH demonstrated the lowest IBS (0.1664, 95% CI: 0.1410–0.1932), indicating the lowest prediction error, while DeepSurv achieved a comparable IBS (0.1737, 95% CI: 0.1527–0.1961). In the internal validation OSLO-COMET dataset, CoxPH retained the highest C-index (0.5933, 95% CI: 0.5003–0.6794), whereas DeepSurv achieved the lowest IBS (0.2043, 95% CI: 0.1776–0.2336). These findings highlight a trade-off between discrimination and prediction error across cohorts. CoxPH consistently achieved the highest C-index on the test set and the independent internal validation set, and lowest IBS on the test set, whereas DeepSurv yielded the lowest IBS in the internal validation set.

To improve predictive performance and mitigate overfitting, all models underwent hyperparameter optimization and regularization. Despite these measures, the performance gains, which were observed with more complex models on the training data did not consistently translate to the test set or the internal validation OSLO-COMET dataset. Overall, the traditional CoxPH model remained competitive and robust regarding C-index across all cohorts. While DeepSurv demonstrated consistently low prediction error as reflected by IBS values across all cohorts.

No pairwise comparison between the six models reached statistical significance after Holm Bonferroni correction for multiple comparisons (all p_holm ≥ 0.05) (**Supplementary Table 2**) for either discrimination, assessed using the C-index, or prediction error, assessed using the IBS, in either the test set or the internal validation set. In the test set, six pairwise comparisons of prediction error showed nominal uncorrected p-values below 0.05: CoxPH versus DeepHit (p = 0.014), CoxPH versus XGBoost (p = 0.018), CoxPH versus RSF (p = 0.020), DeepSurv versus DeepHit (p = 0.026), XGBoost versus DeepSurv (p = 0.030), and CoxPH versus SVM (p = 0.048). These nominal findings consistently favored CoxPH and DeepSurv in terms of lower prediction error, with XGBoost and DeepHit showing comparatively higher prediction error. However, none survived correction for multiple comparisons (all p_holm ≥ 0.84). Overall, the six modeling approaches demonstrated statistically comparable performance across both the test set and the internal validation dataset.

The results of the SHAP-based explainability analyses for the CoxPH and DeepSurv models: these two models demonstrated the most consistent overall predictive performance, while CoxPH achieved the highest discrimination, as measured by the C-index, DeepSurv exhibited the lowest prediction error, as reflected by the lowest IBS, on both the test set and the internal validation OSLO-COMET dataset. The SHAP plots for both models are presented in **Figure 3**, illustrating the contribution of individual preoperative features for survival predictions across the two evaluation cohorts.

**Figure 3:**
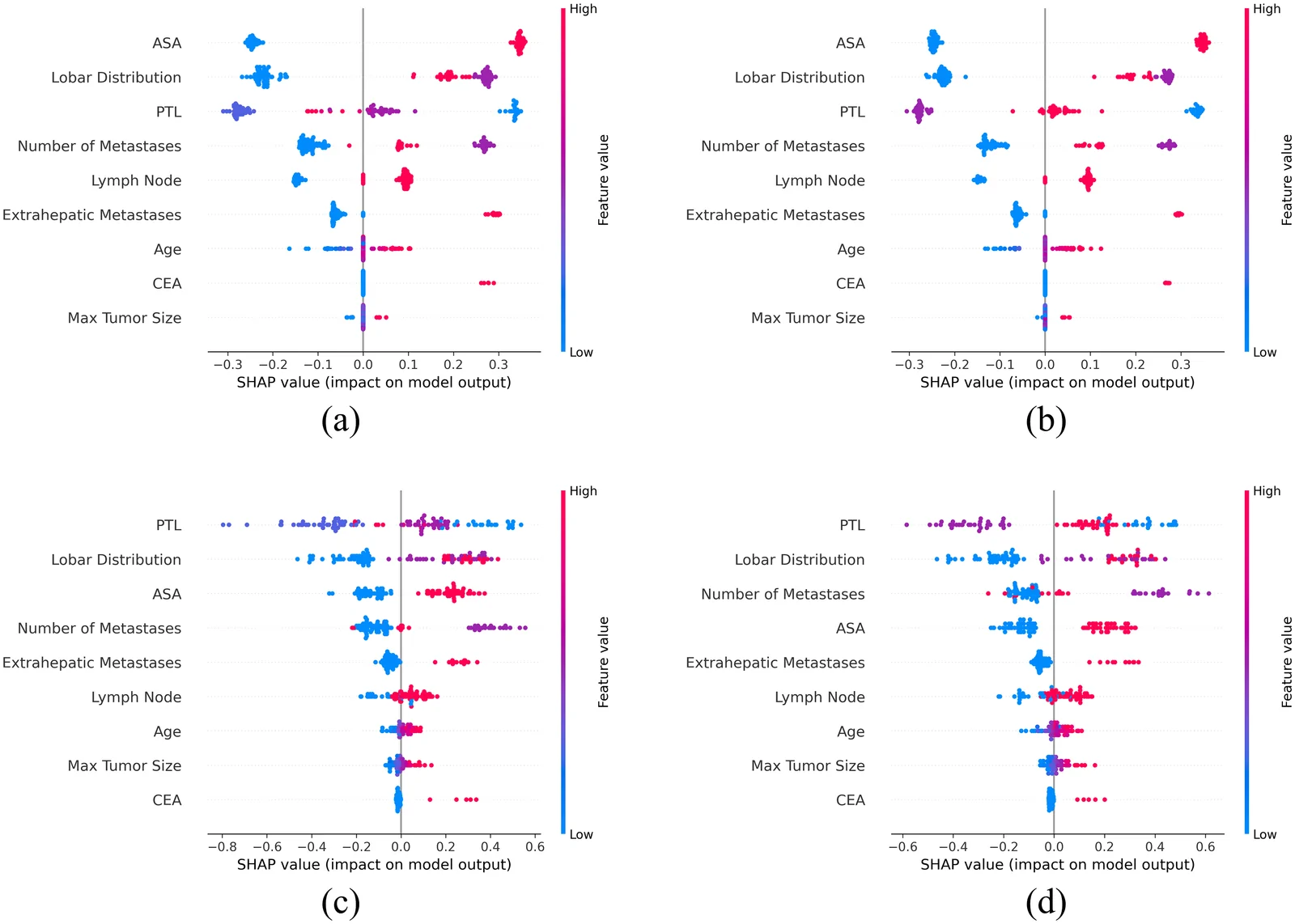
SHAP-based explainability of CoxPH and DeepSurv models on the held-out test set and on the independent internal validation cohort. SHAP-based explainability analysis of CoxPH and DeepSurv models. **(a)** SHAP-based explainability of CoxPH model on the test set, **(b)** SHAP-based explainability of CoxPH model on the internal validation set, **(c)** SHAP-based explainability of DeepSurv model on the test set, and **(d)** SHAP-based explainability of DeepSurv model on the internal validation set. ASA-American Society of Anesthesiologists; PTL-Primary Tumor Location, CEA-Carcinoembryonic Antigen.

Across all models and datasets, the top four most influential features were consistently ASA score, lobar distribution, PTL, and number of metastases, indicating that these variables had the strongest impact on predicting survival outcomes.

For the CoxPH model, SHAP analysis on the test set revealed the following patterns: Higher ASA, right-sided PTL and metastases located in the left lobe of the liver were associated with higher risk. Age, presence of extrahepatic metastases, number of metastases, tumor size, CEA level and lymph node status were also associated with high risk.

Similar patterns were observed in the CoxPH internal validation OSLO-COMET set, with ASA, lobar distribution, PTL and number of metastases again ranking highest, confirming the robustness of these feature contributions across datasets.

For the DeepSurv model, SHAP analysis showed largely consistent results with CoxPH: Higher ASA, right-sided PTL, lobar distribution and number of metastases remained the top contributors for high risk on both datasets. Other features, including extrahepatic metastases, age, maximum tumor size, and CEA, contributed to risk predictions but with lower magnitude compared to the top four variables.

Overall, the SHAP analysis highlights that ASA score, lobar distribution, PTL, and number of metastases are the dominant preoperative predictors of survival across both machine-learning and deep-learning models, as shown in **Figure 3**.

## Discussion

In this study, we developed and compared multiple machine-learning and deep-learning survival models for patients undergoing liver resection for CRLM using exclusively preoperative clinical parameters. The objective was to evaluate whether these models could provide individualized estimates of 1-, 3-, and 5-year overall survival at the time of preoperative decision-making. The main finding was that the CoxPH model demonstrated the most consistent predictive performance across the test set and the independent internal validation seet derived from the OSLO-COMET trial (31). DeepSurv showed broadly comparable performance, despite the relatively small development dataset, whereas the more complex models did not demonstrate a clear and consistent advantage over CoxPH.

The incorporation of SHAP-based interpretability analyses provided additional insight into the contribution of individual clinical variables to model predictions (**Figure 3**) (7, 8, 36). Variables such as age, ASA score, CEA level, number of liver metastases, presence of extrahepatic disease, and PTL contributed substantially to risk estimation. The overall consistency of the most influential predictors between CoxPH and DeepSurv is noteworthy, as these variables are also well-established prognostic factors in patients with CRLM. The agreement between model-derived predictors and established clinical knowledge supports the face validity of the models and demonstrates the potential usefulness of interpretable machine-learning approaches in this setting. However, SHAP-based feature importance should not be interpreted as evidence of causal relationships or as independent confirmation of prognostic effects.

Across the models, discrimination in the OSLO-COMET internal validation set was modest, with C-indices ranging from approximately 0.56 to 0.59. These values indicate that the models provided limited-to-moderate discrimination in an independent cohort from the same institution and should therefore not be interpreted as demonstrating clinical readiness. Importantly, CoxPH maintained the highest discrimination in the internal validation set, whereas differences between CoxPH and DeepSurv were relatively small. In the test cohort, CoxPH also demonstrated a lower IBS than DeepSurv (0.16642 vs. 0.17373), indicating lower overall prediction error according to this metric.

The individualized survival estimates generated by CoxPH, DeepSurv, and RSF were broadly comparable with those obtained using the established BPI, which is also based on conventional preoperative clinical variables (**Supplementary Tables 3**–**S5**) (8). However, this comparison should be interpreted cautiously. The present study was not specifically designed or powered to establish superiority or formal equivalence between the machine-learning models and BPI, and the supplementary comparison was primarily exploratory. Therefore, the similarity in predicted survival estimates should be regarded as contextual evidence rather than as a formal demonstration that the proposed models outperform or are equivalent to BPI. Future studies should prospectively compare these approaches using standardized discrimination, calibration, and clinical utility measures within the same patient population.

The similar performance of CoxPH compared with more complex machine-learning approaches may indicate that, when using structured preoperative clinical variables, much of the available prognostic information is captured by established predictors and their relatively simple relationships with outcome. This finding is consistent with the principle that increased model complexity does not necessarily translate into improved predictive performance, particularly when datasets are relatively small. In such settings, simpler models such as CoxPH may offer advantages in interpretability, transparency, and ease of clinical evaluation. Nevertheless, these potential practical advantages should not be equated with demonstrated clinical utility, which requires prospective evaluation and external validation.

The attenuation of predictive performance observed in the OSLO-COMET internal validation cohort may partly reflect differences in baseline characteristics between the development and the internal validation OSLO-COMET set. As shown in **Table 1**, the OSLO-COMET cohort had a lower median CEA level (3.6 µg/L vs. 25.4 µg/L), a smaller proportion of male patients (40% vs. 61%), and a higher proportion of patients with ASA 3 status, together with differences in other tumor- and patient-related characteristics. These differences may have contributed to the reduction in C-index observed between the test set and the OSLO-COMET dataset. Similarly, the increase in IBS observed in the validation cohort suggests greater overall prediction error when the models were applied to a population with different baseline characteristics. However, these observations should be considered hypothesis-generating rather than evidence that specific baseline differences caused the reduction in performance, particularly given the relatively small size of the validation cohort.

The performance of our models can be further contextualized by comparison with previously published prognostic models. Lam et al. (11) developed an AI-based prognostic model using a cohort of 572 patients and reported a C-index of 0.651 following internal validation. Our models demonstrated lower discrimination. However, direct comparison should be made cautiously because the predictor sets and study populations differed substantially. In particular, the model developed by Lam et al. incorporated perioperative and postoperative variables, whereas our model was deliberately restricted to preoperative parameters. This distinction is important because our objective was to estimate survival using information available before surgery, thereby preserving the potential applicability of the model to preoperative counseling and decision-making without relying on postoperative information.

Similarly, Ding et al. (12) reported a prognostic model for patients with CRLM and synchronous metastases following preoperative chemotherapy, achieving a C-index of 0.642 in internal validation and 0.671 in external validation. Their model also incorporated perioperative and postoperative variables and was restricted to patients with synchronous metastases. Consequently, the higher reported C-index cannot be directly compared with the present study. Nevertheless, these studies illustrate that expanding the predictor set and selecting more homogeneous patient populations may improve predictive performance, although potentially at the expense of applicability at the initial preoperative decision point.

A recent systematic review by Kokkinakis et al. (37) highlighted that, despite rapid advances in artificial intelligence, substantial challenges remain regarding predictive accuracy, validation, and clinical utility in CRLM prognostication. Our findings are consistent with this broader literature and emphasize that methodological complexity alone is unlikely to overcome limitations related to sample size, predictor availability, and cohort heterogeneity.

### Limitations and future perspectives

Several limitations should be acknowledged. First, the development cohort was derived from a single high-volume tertiary center, limiting the generalizability of the findings. Although the OSLO-COMET cohort was independent of the development cohort and provided an independent internal validation dataset, it originated from the same institution and therefore does not constitute true external validation. The relatively modest discrimination observed in this cohort further emphasizes the need for validation in larger, geographically and demographically distinct multicenter populations before any conclusions regarding broad applicability or clinical implementation can be made.

Second, the relatively small development dataset limits the ability of deep-learning architectures to exploit complex nonlinear relationships. Only 245 patients were available for model development, which is a considerable constraint for the development and tuning of neural-network-based survival models. The comparable performance of DeepSurv to CoxPH should therefore be interpreted cautiously and should not be taken as evidence that deep-learning approaches are inherently effective in small clinical datasets. Larger multicenter datasets will be necessary to determine whether the potential advantages of nonlinear modeling can be realized in this setting. Synthetic data generation may represent one possible future strategy for addressing data limitations, although such approaches require careful validation to ensure that they do not introduce artificial patterns or distort clinically relevant relationships (38).

Third, the absence of preoperative radiological and radiomic features represents an important limitation. The present models were intentionally restricted to routinely available preoperative clinical variables to preserve applicability at the time of treatment planning. However, radiological tumor characteristics may contain additional prognostic information that is not captured by conventional clinical variables. Future studies should therefore investigate multimodal models combining clinical, radiological, and potentially molecular information.

Fourth, the categorization of predicted survival probabilities into 1-, 3-, and 5-year prediction groups should be interpreted cautiously because universally accepted thresholds for defining clinically meaningful prediction accuracy have not been established. Such categorizations are therefore descriptive rather than validated clinical decision thresholds.

Despite these limitations, the present framework provides a basis for future investigation of preoperative survival prediction in CRLM. Larger multicenter cohorts should be used to externally validate the models and to assess discrimination, calibration, and clinical utility prospectively. Integration of additional preoperative biomarkers, radiomic features, and histopathologic growth patterns where available, may provide complementary prognostic information. Such multimodal approaches could potentially improve individualized risk estimation while preserving the important requirement that relevant information be available before treatment decisions are made.

## Conclusion

In conclusion, CoxPH demonstrated the most consistent performance across test and internal validation cohorts, while DeepSurv showed comparable performance despite the limited development sample. SHAP analyses identified predictors consistent with established clinical prognostic factors. However, modest discrimination, the lack of true external validation, and limited sample size preclude conclusions regarding clinical implementation. These findings suggest that greater model complexity does not necessarily improve prognostic performance over conventional CoxPH when using structured preoperative clinical data. Larger multicenter studies incorporating imaging and molecular features, calibration, and clinical utility assessment are needed to establish the potential of these approaches for individualized preoperative prognostication in CRLM.

## Data Availability

The raw data supporting the conclusions of this article will be made available by the authors, without undue reservation.
