# Supplementary material for: Explainable AI–based prognostication of patients with resectable colorectal liver metastases using preoperative clinical parameters: is it comparable to traditional clinical scoring systems?
Source: Front Oncol. 2026 Sep 21;16:1890766. doi: 10.3389/fonc.2026.1890766 (PMC13634838; doi:10.3389/fonc.2026.1890766)
Supplement: Supplementary file 1 [file DataSheet1.pdf]

Table S1. Univariate analysis for overall survival on training set after Imputation

Variable	n_used	Hazard Ratio (HR)	Lower 95 % CI	Upper 95 % CI	P-value	Global P-value
Age	245	1.0445	1.0236	1.0658	**0.0002**	**-**
Max tumor size	245	1.0118	1.0034	1.0204	**0.0058**	**-**
ASA	245	2.2014	1.5035	3.2233	**<0.0001**	**-**
CEA	245	2.6018	1.3538	5.0002	**0.0041**	**-**
T-status	245	0.9760	0.5568	1.7104	0.9322	-
Gender	245	0.8333	0.5655	1.2278	0.3563	-
Previous laparoscopic surgery	245	0.8775	0.6012	1.2810	0.4985	-
Previous open surgery	245	1.0485	0.7199	1.5271	0.8050	-
Extrahepatic metastasis	245	1.9246	1.2463	2.9721	**0.0031**	**-**
Lymph node involvement in the primary tumor	245	1.5307	1.0345	2.2648	**0.0331**	**-**
Liver metastasis diagnosis (synchronous)	245	1.4186	0.9324	2.1583	0.1024	-
Previous abdominal surgery	245	0.6421	0.2036	2.0245	0.4496	-
Neoadjuvant chemotherapy	245	1.3165	0.9054	1.9143	0.1500	-

Number of metastases_1	245	ref	ref	ref	ref	**0.0448**
Number of metastases_2		1.7520	1.1401	2.6924	0.0105	
Number of metastases_3		1.2673	0.7559	2.1249	0.3690	
*Repeated laparoscopy_0	245	ref	ref	ref	ref	0.6863
Repeated laparoscopy_1		1.2762	0.7152	2.2770	0.4091	
Repeated laparoscopy_2		0.7454	0.1038	5.3507	0.7701	
Dukes	245					0.2006
A		ref	ref	ref	ref	
B		0.9049	0.2101	3.8983	0.8934	
C		1.3847	0.3287	5.8326	0.6573	
D		1.5359	0.3744	6.3005	0.5513	
PTL	245					**0.0301**
Right colon		ref	ref	ref	ref	
Left colon		0.4596	0.2841	0.7435	0.0015	
Rectum		0.7420	0.4700	1.7114	0.2002	
Transversum		0.5221	0.1605	1.6980	0.2801	
Multiple		0.5792	0.1782	1.8832	0.3640	
Lobar distribution	245					**0.0120**
Right		ref	ref	ref	ref	
Left		1.7841	1.1614	2.7404	0.0082	

Both		1.7503	1.0831	2.8282	0.0222	

ASA = American Society of Anesthesiologists; CEA = Carcinoembryonic Antigen; PTL = Primary Tumor Location

Table S2. Pairwise Performance Comparisons Across Six Survival Models on the Test and on the Internal Validation Set

Dataset	Metric	Model_a	Model_b	p_value	p_holm
Test_Set	C-index	CoxPH	RSF	0.63	1
Test_Set	IBS	CoxPH	RSF	0.02	1
Test_Set	C-index	CoxPH	SVM	0.142	1
Test_Set	IBS	CoxPH	SVM	0.048	1
Test_Set	C-index	CoxPH	XGBoost	0.286	1
Test_Set	IBS	CoxPH	XGBoost	0.018	1
Test_Set	C-index	CoxPH	DeepSurv	0.342	1
Test_Set	IBS	CoxPH	DeepSurv	0.642	1
Test_Set	C-index	CoxPH	DeepHit	0.314	1
Test_Set	IBS	CoxPH	DeepHit	0.014	0.84
Test_Set	C-index	RSF	SVM	0.472	1
Test_Set	IBS	RSF	SVM	0.858	1
Test_Set	C-index	RSF	XGBoost	0.304	1
Test_Set	IBS	RSF	XGBoost	0.268	1
Test_Set	C-index	RSF	DeepSurv	0.898	1
Test_Set	IBS	RSF	DeepSurv	0.062	1
Test_Set	C-index	RSF	DeepHit	0.64	1
Test_Set	IBS	RSF	DeepHit	0.624	1
Test_Set	C-index	SVM	XGBoost	0.914	1
Test_Set	IBS	SVM	XGBoost	0.626	1
Test_Set	C-index	SVM	DeepSurv	0.462	1
Test_Set	IBS	SVM	DeepSurv	0.148	1
Test_Set	C-index	SVM	DeepHit	0.894	1
Test_Set	IBS	SVM	DeepHit	0.794	1
Test_Set	C-index	XGBoost	DeepSurv	0.558	1
Test_Set	IBS	XGBoost	DeepSurv	0.03	1
Test_Set	C-index	XGBoost	DeepHit	0.798	1
Test_Set	IBS	XGBoost	DeepHit	0.766	1
Test_Set	C-index	DeepSurv	DeepHit	0.644	1
Test_Set	IBS	DeepSurv	DeepHit	0.026	1

Internal_Validation	C-index	CoxPH	RSF	0.666	1
Internal_Validation	IBS	CoxPH	RSF	0.338	1
Internal_Validation	C-index	CoxPH	SVM	0.968	1
Internal_Validation	IBS	CoxPH	SVM	0.408	1
Internal_Validation	C-index	CoxPH	XGBoost	0.574	1
Internal_Validation	IBS	CoxPH	XGBoost	0.536	1
Internal_Validation	C-index	CoxPH	DeepSurv	0.736	1
Internal_Validation	IBS	CoxPH	DeepSurv	0.82	1
Internal_Validation	C-index	CoxPH	DeepHit	0.49	1
Internal_Validation	IBS	CoxPH	DeepHit	0.422	1
Internal_Validation	C-index	RSF	SVM	0.76	1
Internal_Validation	IBS	RSF	SVM	0.95	1
Internal_Validation	C-index	RSF	XGBoost	0.66	1
Internal_Validation	IBS	RSF	XGBoost	0.96	1
Internal_Validation	C-index	RSF	DeepSurv	0.924	1
Internal_Validation	IBS	RSF	DeepSurv	0.522	1
Internal_Validation	C-index	RSF	DeepHit	0.714	1
Internal_Validation	IBS	RSF	DeepHit	0.838	1
Internal_Validation	C-index	SVM	XGBoost	0.568	1
Internal_Validation	IBS	SVM	XGBoost	0.978	1
Internal_Validation	C-index	SVM	DeepSurv	0.804	1
Internal_Validation	IBS	SVM	DeepSurv	0.588	1
Internal_Validation	C-index	SVM	DeepHit	0.552	1
Internal_Validation	IBS	SVM	DeepHit	0.89	1
Internal_Validation	C-index	XGBoost	DeepSurv	0.728	1
Internal_Validation	IBS	XGBoost	DeepSurv	0.668	1
Internal_Validation	C-index	XGBoost	DeepHit	0.932	1
Internal_Validation	IBS	XGBoost	DeepHit	0.856	1
Internal_Validation	C-index	DeepSurv	DeepHit	0.626	1
Internal_Validation	IBS	DeepSurv	DeepHit	0.452	1

Table S3. 1, 3 and 5-Year Survival Predictions for the 88 Patients from OSLO-COMET Dataset: BPI versus CoxPH versus DeepSurv versus RSF

		BPI Predictions				CoxPH Predictions			DeepSurv Predictions			RSF Predictions		
	Actual survival by months	BPI Score	1-year %	3-year %	5-year %	1-year %	3-year %	5-year %	1-year %	3-year %	5-year %	1-year %	3-year %	5-year %
Patient 1	80	6	90	62	43	95	70	50	96	76	59	94	59	43
Patient 2	119	12	81	46	28	98	89	80	98	86	74	99	94	91
Patient 3	6	9	90	62	43	90	50	25	92	55	39	87	48	25
Patient 4	39	7	90	62	43	95	72	53	97	80	64	94	72	55
Patient 5	36	0	97	82	66	99	95	84	98	89	80	99	97	96
Patient 6	45	14	81	46	28	96	76	58	95	72	53	93	80	50
Patient 7	22	5	93	69	51	96	75	57	93	61	39	95	59	41
Patient 8	115	5	93	69	51	96	78	62	97	78	62	98	86	69
Patient 9	14	5	93	69	51	96	74	56	94	62	40	98	79	69
Patient 10	25	3	95	76	59	97	81	66	94	63	41	97	79	62
Patient 11	61	3	95	76	59	96	78	62	96	73	54	94	81	69
Patient 12	114	5	93	69	51	98	86	75	98	86	75	97	86	81
Patient 13	37	5	93	69	51	97	79	63	97	78	62	97	87	77
Patient 14	74	7	90	62	43	98	90	81	98	88	78	97	83	74
Patient 15	111	2	95	76	59	95	70	49	94	63	41	95	65	52
Patient 16	34	5	93	69	51	98	88	78	98	85	73	99	93	87
Patient 17	111	8	90	62	43	90	47	22	93	58	35	97	50	36
Patient 18	110	5	93	69	51	96	75	58	93	61	39	95	74	52
Patient 19	40	5	93	69	51	94	64	42	96	75	58	95	73	60
Patient 20	16	5	93	69	51	97	79	63	96	73	55	96	89	71
Patient 21	110	0	97	82	66	97	83	70	96	77	61	98	92	79
Patient 22	108	5	93	69	51	97	83	69	97	81	67	99	89	72
Patient 23	100	7	90	62	43	94	64	41	95	71	52	81	62	50
Patient 24	48	5	93	69	51	96	74	56	94	62	40	98	71	58
Patient 25	26	6	90	62	43	96	76	60	98	85	73	93	61	44
Patient 26	25	5	93	69	51	97	81	67	98	86	75	99	90	75
Patient 27	102	2	95	76	59	97	84	71	97	83	69	98	82	74
Patient 28	16	12	81	46	28	89	43	19	93	59	36	88	46	16
Patient 29	101	3	95	76	59	98	87	76	97	82	68	99	91	70
Patient 30	24	2	95	76	59	95	70	50	92	54	31	91	44	29
Patient 31	78	3	95	76	59	96	74	55	96	72	54	98	75	56
Patient 32	99	3	95	76	59	96	77	60	96	77	60	99	89	71
Patient 33	33	5	93	69	51	96	78	62	97	80	65	98	79	58
Patient 34	98	3	95	76	59	61	50	26	92	56	33	88	47	19
Patient 35	11	9	90	62	43	93	59	35	92	57	33	97	59	34
Patient 36	95	2	95	76	59	95	71	51	95	69	48	94	67	48
Patient 37	26	5	93	69	51	94	67	46	96	79	61	96	83	76
Patient 38	95	5	93	69	51	99	91	83	98	89	79	99	96	93
Patient 39	94	10	86	54	35	93	61	39	95	71	52	95	48	23
Patient 40	44	5	93	69	51	94	65	43	95	68	47	97	70	43
Patient 41	33	5	93	69	51	90	47	23	89	44	20	94	44	26

Patient 42	86	3	95	76	59	89	44	20	88	41	18	95	46	17
Patient 43	12	12	81	46	28	86	34	12	89	43	19	87	39	19
Patient 44	86	0	97	82	66	96	76	59	93	62	40	97	68	49
Patient 45	32	7	90	62	43	94	67	45	96	78	61	99	68	43
Patient 46	20	7	90	62	43	97	81	66	98	86	75	99	90	75
Patient 47	40	14	81	46	28	95	71	50	95	67	47	93	74	39
Patient 48	92	3	95	76	59	99	91	83	98	88	79	99	96	91
Patient 49	92	3	95	76	59	99	91	84	98	89	80	99	94	92
Patient 50	91	3	95	76	59	94	65	43	93	61	39	93	53	35
Patient 51	86	3	95	76	59	96	76	58	96	74	57	98	67	43
Patient 52	74	5	93	69	51	96	76	58	96	75	57	99	69	46
Patient 53	42	7	90	62	43	96	74	55	94	62	40	98	73	60
Patient 54	88	13	81	46	28	86	35	13	90	48	24	90	53	34
Patient 55	34	5	93	69	51	92	55	31	92	57	34	98	58	39
Patient 56	87	7	90	62	43	96	78	61	96	75	58	99	82	67
Patient 57	87	5	93	69	51	96	77	59	97	80	63	97	58	33
Patient 58	14	11	81	46	28	96	74	56	96	77	60	90	67	50
Patient 59	19	9	90	62	43	92	57	33	90	46	22	94	62	47
Patient 60	86	5	93	69	51	93	59	35	93	58	35	98	71	60
Patient 61	21	2	95	76	59	94	64	41	96	75	58	95	71	58
Patient 62	86	5	93	69	51	96	76	59	96	76	59	97	79	62
Patient 63	68	7	90	62	43	91	51	27	92	54	31	94	52	37
Patient 64	84	9	90	62	43	96	76	60	96	76	59	94	57	37
Patient 65	81	5	93	69	51	95	70	50	91	49	25	99	71	61
Patient 66	49	5	93	69	51	95	69	49	96	73	55	98	76	58
Patient 67	72	12	81	46	28	96	73	54	97	78	61	93	80	55
Patient 68	14	12	81	46	28	87	39	16	91	52	29	85	45	20
Patient 69	52	7	90	62	43	94	66	45	96	77	60	99	69	44
Patient 70	79	7	90	62	43	93	60	36	92	53	29	99	54	41
Patient 71	79	12	81	46	28	93	63	40	94	63	41	93	76	60
Patient 72	42	6	90	62	43	93	63	40	94	64	43	94	61	44
Patient 73	41	2	95	76	59	96	75	56	97	81	67	96	84	69
Patient 74	15	5	93	69	51	90	48	24	89	42	19	98	53	31
Patient 75	50	14	81	46	28	94	65	43	95	70	51	96	74	43
Patient 76	77	5	93	69	51	96	75	57	96	75	58	94	57	42
Patient 77	52	4	95	76	59	93	61	38	96	72	54	94	47	26
Patient 78	41	7	90	62	43	98	88	78	98	85	73	99	93	86
Patient 79	29	5	93	69	51	96	77	59	96	76	58	94	68	48
Patient 80	74	5	93	69	51	97	82	68	98	86	75	99	90	77
Patient 81	74	3	95	76	59	97	84	71	98	89	80	99	88	80
Patient 82	40	5	93	69	51	93	63	40	95	68	47	95	56	27
Patient 83	46	5	93	69	51	92	57	34	93	57	34	93	50	22
Patient 84	50	4	95	76	59	98	89	80	98	85	74	99	93	90
Patient 85	59	5	93	69	51	90	49	25	90	47	23	94	46	23
Patient 86	72	4	95	76	59	93	63	40	96	76	59	93	54	35

Patient 87	72	0	97	82	66	96	75	57	95	72	53	98	67	49
Patient 88	72	3	95	76	59	98	87	76	98	83	70	99	93	79

BPI = Basingstoke Predictive Index; CoxPH = Cox Proportional Hazard; RSF = Random Survival Forest

Table S4. 1, 3 and 5-Year Survival Predictions for the 245 Training-Set Patients: BPI versus CoxPH versus DeepSurv versus RSF

		BPI Predictions				CoxPH Predictions			DeepSurv Predictions			RSF Predictions		
	Actual survival by months	BPI Score	1-year %	3-year %	5-year %	1-year %	3-year %	5-year %	1-year %	3-year %	5-year %	1-year %	3-year %	5-year %
Patient 1	79	0	97	82	66	99	93	86	99	90	82	99	90	84
Patient 2	79	3	95	76	59	99	91	83	98	89	79	99	98	95
Patient 3	79	5	93	69	51	97	79	63	97	78	63	97	84	73
Patient 4	8	14	81	46	28	97	79	64	96	77	60	81	67	49
Patient 5	72	5	93	69	51	97	80	64	97	79	63	97	92	83
Patient 6	68	3	95	76	59	94	65	43	91	51	27	98	72	60
Patient 7	27	18	68	31	17	84	30	9	90	48	24	92	48	25
Patient 8	79	3	95	76	59	94	67	46	96	72	53	95	79	62
Patient 9	40	11	81	46	28	97	79	63	96	74	56	96	79	52
Patient 10	37	8	90	62	43	95	71	51	96	75	57	96	68	40
Patient 11	41	5	93	69	51	97	80	64	98	89	79	99	90	81
Patient 12	30	5	93	69	51	92	55	31	93	59	36	95	53	22
Patient 13	60	15	75	38	21	93	62	39	94	62	40	92	73	56
Patient 14	49	14	81	46	28	93	61	38	95	67	46	93	75	36
Patient 15	78	5	93	69	51	96	77	60	96	77	61	99	87	71
Patient 16	68	5	93	69	51	97	84	70	97	82	68	97	87	67
Patient 17	77	3	95	76	59	97	78	62	98	85	74	99	74	60
Patient 18	76	5	93	69	51	96	75	57	96	75	57	89	65	52
Patient 19	45	7	90	62	43	97	81	66	98	86	74	98	85	65
Patient 20	33	9	90	62	43	96	78	61	98	87	76	97	73	60
Patient 21	18	12	81	46	28	89	43	19	92	55	31	96	61	33
Patient 22	20	12	81	46	28	96	77	59	97	80	65	94	63	38
Patient 23	73	5	93	69	51	95	72	53	95	70	50	94	69	52
Patient 24	13	5	93	69	51	95	70	49	95	67	46	96	50	33
Patient 25	73	9	90	62	43	97	80	65	97	79	63	98	87	79
Patient 26	20	5	93	69	51	90	48	24	90	46	22	95	36	22
Patient 27	72	12	81	46	28	95	72	53	96	74	57	92	80	65
Patient 28	72	5	93	69	51	97	83	69	98	87	76	99	87	73
Patient 29	72	7	90	62	43	98	89	79	98	85	74	99	92	87
Patient 30	72	7	90	62	43	98	85	73	98	85	73	98	90	79
Patient 31	72	7	90	62	43	95	72	53	97	80	65	96	77	58
Patient 32	11	5	93	69	51	92	55	31	90	47	24	83	53	38
Patient 33	71	5	93	69	51	98	87	75	98	90	81	99	88	77
Patient 34	71	2	95	76	59	97	80	65	98	85	74	98	86	65

Patient 35	5	19	68	31	17	88	40	17	92	55	32	73	44	24
Patient 36	71	9	90	62	43	96	73	54	96	75	57	94	81	69
Patient 37	29	14	81	46	28	88	41	18	92	54	30	91	49	11
Patient 38	7	10	86	54	35	96	74	56	96	76	59	65	48	38
Patient 39	71	5	93	69	51	92	56	32	90	48	24	90	73	57
Patient 40	7	16	68	31	17	86	35	13	88	39	17	46	20	12
Patient 41	56	11	81	46	28	94	64	42	93	59	36	94	65	41
Patient 42	42	3	95	76	59	98	86	74	97	81	66	98	79	59
Patient 43	70	14	81	46	28	97	84	70	97	78	62	96	85	70
Patient 44	26	9	90	62	43	98	88	77	98	84	72	98	75	62
Patient 45	47	10	86	54	35	94	66	44	93	57	34	90	70	47
Patient 46	69	5	93	69	51	96	73	54	97	80	66	96	75	60
Patient 47	69	3	95	76	59	97	79	63	95	70	51	96	88	73
Patient 48	69	12	81	46	28	96	73	53	96	74	56	93	79	67
Patient 49	49	12	81	46	28	95	72	53	95	68	48	94	77	45
Patient 50	69	9	90	62	43	97	79	62	96	77	61	98	86	78
Patient 51	69	0	97	82	66	97	81	65	95	68	47	99	85	79
Patient 52	68	5	93	69	51	98	88	77	98	85	73	99	88	82
Patient 53	68	5	93	69	51	96	76	59	95	71	52	94	84	70
Patient 54	32	5	93	69	51	86	34	12	88	40	17	92	32	13
Patient 55	12	4	95	76	59	93	58	35	93	60	38	97	51	38
Patient 56	67	14	81	46	28	97	81	67	96	77	60	93	84	61
Patient 57	40	7	90	62	43	94	64	41	93	58	35	93	73	50
Patient 58	20	3	95	76	59	95	72	52	93	61	39	91	35	22
Patient 59	15	8	90	62	43	97	83	69	96	76	58	93	55	35
Patient 60	50	5	93	69	51	97	82	68	98	87	76	99	82	42
Patient 61	66	3	95	76	59	96	77	59	96	76	59	99	74	56
Patient 62	29	7	90	62	43	95	70	50	96	77	60	99	74	57
Patient 63	15	2	95	76	59	93	61	37	94	63	41	98	47	29
Patient 64	50	7	90	62	43	89	44	20	91	49	25	96	52	26
Patient 65	36	5	93	69	51	92	55	31	92	57	33	94	49	13
Patient 66	65	0	97	82	66	98	89	79	98	86	74	99	89	74
Patient 67	56	3	95	76	59	97	83	70	96	74	56	95	81	52
Patient 68	65	4	95	76	59	98	88	78	98	85	72	99	92	85
Patient 69	34	5	93	69	51	90	48	24	89	42	19	98	53	31
Patient 70	63	2	95	76	59	99	91	83	98	89	79	99	91	86
Patient 71	53	13	81	46	28	97	80	65	96	72	53	94	74	51
Patient 72	64	5	93	69	51	98	89	79	98	85	73	99	96	91
Patient 73	62	6	90	62	43	94	63	40	95	70	50	94	74	60
Patient 74	58	3	95	76	59	98	87	76	97	82	69	99	89	63
Patient 75	62	0	97	82	66	94	63	40	91	52	29	90	66	50
Patient 76	6	3	95	76	59	91	54	29	92	56	33	93	62	48
Patient 77	1	3	95	76	59	96	74	55	95	68	48	76	53	29
Patient 78	62	4	95	76	59	98	89	79	98	85	73	99	94	90
Patient 79	62	5	93	69	51	97	83	70	97	82	67	99	89	68

Patient 80	32	3	95	76	59	97	81	67	99	91	83	98	68	58
Patient 81	33	5	93	69	51	96	77	59	94	62	39	98	74	65
Patient 82	14	11	81	46	28	96	76	58	96	75	57	92	45	27
Patient 83	35	0	97	82	66	96	77	60	96	76	60	99	68	52
Patient 84	61	7	90	62	43	97	82	67	98	87	77	95	80	64
Patient 85	61	3	95	76	59	97	80	65	98	89	80	99	88	80
Patient 86	14	4	95	76	59	90	49	25	90	46	23	95	38	24
Patient 87	60	2	95	76	59	98	88	78	98	85	73	99	94	85
Patient 88	60	2	95	76	59	93	59	35	93	59	36	98	72	61
Patient 89	32	7	90	62	43	94	65	43	93	61	38	91	48	30
Patient 90	59	5	93	69	51	98	84	72	97	83	69	99	93	82
Patient 91	35	5	93	69	51	94	65	43	96	77	60	95	77	68
Patient 92	59	7	90	62	43	96	74	55	97	83	70	99	83	70
Patient 93	59	7	90	62	43	94	66	44	96	77	60	95	66	42
Patient 94	53	3	95	76	59	95	69	48	96	73	55	99	68	39
Patient 95	58	5	93	69	51	97	79	63	98	88	79	99	84	75
Patient 96	58	5	93	69	51	93	59	36	92	56	33	93	76	63
Patient 97	58	5	93	69	51	96	74	55	94	62	40	97	71	57
Patient 98	57	5	93	69	51	93	58	34	92	55	31	93	72	58
Patient 99	57	5	93	69	51	96	74	55	97	79	63	99	88	76
Patient 100	56	3	95	76	59	97	82	68	98	87	77	99	88	80
Patient 101	55	5	93	69	51	96	76	58	97	81	66	96	83	69
Patient 102	54	18	68	31	17	94	63	40	96	76	58	91	55	35
Patient 103	54	3	95	76	59	99	90	82	98	88	79	99	93	87
Patient 104	54	10	86	54	35	97	81	66	97	79	63	95	81	70
Patient 105	54	0	97	82	66	97	78	61	97	78	63	99	90	77
Patient 106	54	3	95	76	59	98	87	76	97	83	69	98	90	72
Patient 107	54	10	86	54	35	89	44	20	89	42	19	88	51	29
Patient 108	53	5	93	69	51	97	83	70	97	82	68	99	91	76
Patient 109	48	3	95	76	59	90	46	22	89	45	21	95	36	17
Patient 110	43	15	75	38	21	92	55	31	93	60	37	86	55	22
Patient 111	30	3	95	76	59	93	59	36	91	52	28	94	35	22
Patient 112	17	5	93	69	51	95	70	49	91	51	27	96	61	40
Patient 113	52	5	93	69	51	94	67	46	97	78	62	96	83	77
Patient 114	37	3	95	76	59	96	78	61	95	68	47	97	66	47
Patient 115	52	5	93	69	51	98	90	81	98	88	77	93	65	51
Patient 116	29	14	81	46	28	95	72	52	96	74	56	89	71	51
Patient 117	23	5	93	69	51	98	88	77	98	84	71	99	90	76
Patient 118	51	2	95	76	59	97	82	68	98	87	76	99	90	75
Patient 119	51	3	95	76	59	97	84	70	98	88	78	99	94	84
Patient 120	29	5	93	69	51	94	66	45	90	49	25	99	47	32
Patient 121	50	5	93	69	51	96	74	55	97	79	63	99	80	66
Patient 122	50	3	95	76	59	98	84	71	97	78	61	98	92	80
Patient 123	50	9	90	62	43	94	66	44	95	68	48	94	82	64
Patient 124	50	2	95	76	59	96	77	60	97	81	67	95	86	74

Patient 125	50	7	90	62	43	94	67	45	94	65	44	99	70	47
Patient 126	17	8	90	62	43	96	77	61	96	76	58	96	56	42
Patient 127	22	7	90	62	43	94	67	45	91	49	26	98	79	68
Patient 128	13	5	93	69	51	96	77	59	97	81	67	96	46	37
Patient 129	49	3	95	76	59	95	72	52	92	56	32	95	70	56
Patient 130	49	2	95	76	59	95	70	50	96	77	61	96	77	67
Patient 131	49	5	93	69	51	94	67	45	97	78	62	96	82	73
Patient 132	9	14	81	46	28	93	59	35	92	54	30	87	66	47
Patient 133	49	8	90	62	43	95	69	48	97	80	65	98	81	62
Patient 134	49	3	95	76	59	96	73	53	94	66	45	97	86	77
Patient 135	49	5	93	69	51	97	82	68	98	86	75	99	90	76
Patient 136	49	3	95	76	59	99	91	83	98	89	80	99	95	94
Patient 137	49	9	90	62	43	98	84	71	98	89	79	96	80	67
Patient 138	20	12	81	46	28	89	44	20	92	53	30	96	43	21
Patient 139	33	7	90	62	43	95	72	53	96	76	58	99	78	60
Patient 140	48	5	93	69	51	97	80	64	97	81	66	99	90	79
Patient 141	48	10	86	54	35	93	62	39	95	68	48	98	69	52
Patient 142	48	2	95	76	59	94	65	43	97	78	62	95	66	52
Patient 143	19	5	93	69	51	94	63	40	94	62	39	97	62	41
Patient 144	47	9	90	62	43	96	74	56	97	78	62	95	83	67
Patient 145	47	7	90	62	43	93	60	36	93	58	35	92	59	32
Patient 146	47	5	93	69	51	98	85	72	97	83	70	99	91	79
Patient 147	47	5	93	69	51	94	67	45	92	56	33	95	70	55
Patient 148	47	5	93	69	51	96	73	54	96	77	60	99	79	63
Patient 149	14	12	81	46	28	94	63	40	96	76	59	97	41	18
Patient 150	32	3	95	76	59	95	70	49	95	68	47	94	53	34
Patient 151	45	12	81	46	28	88	41	17	92	56	32	87	50	11
Patient 152	22	5	93	69	51	91	50	25	92	56	33	97	54	24
Patient 153	46	5	93	69	51	97	81	66	97	81	66	99	90	80
Patient 154	45	5	93	69	51	95	70	49	96	73	55	99	70	43
Patient 155	44	14	81	46	28	91	54	29	95	67	46	96	66	42
Patient 156	44	3	95	76	59	95	71	52	96	74	56	99	84	68
Patient 157	44	3	95	76	59	98	84	72	98	89	79	98	89	78
Patient 158	3	12	81	46	28	92	54	30	95	70	50	85	60	38
Patient 159	43	0	97	82	66	95	68	46	98	84	72	99	70	58
Patient 160	42	7	90	62	43	97	82	67	98	86	75	99	88	75
Patient 161	8	5	93	69	51	91	52	28	90	48	24	78	42	23
Patient 162	18	12	81	46	28	95	70	49	95	71	52	97	83	55
Patient 163	41	3	95	76	59	97	83	69	98	88	78	98	86	72
Patient 164	33	7	90	62	43	98	85	72	97	83	70	98	57	46
Patient 165	41	5	93	69	51	95	71	51	95	70	51	96	71	58
Patient 166	41	3	95	76	59	98	85	72	98	88	78	99	87	79
Patient 167	18	7	90	62	43	97	84	70	97	82	68	97	88	68
Patient 168	18	3	95	76	59	98	89	79	98	86	75	99	91	81
Patient 169	18	5	93	69	51	94	63	40	96	75	57	95	52	31

Patient 170	18	5	93	69	51	91	53	29	92	56	33	98	52	32
Patient 171	11	4	95	76	59	96	78	61	94	66	44	69	56	44
Patient 172	98	7	90	62	43	97	80	65	97	79	63	98	84	76
Patient 173	98	3	95	76	59	95	71	50	92	54	30	97	64	46
Patient 174	98	3	95	76	59	99	91	83	98	89	79	99	98	96
Patient 175	15	10	86	54	35	90	47	23	89	42	19	86	61	46
Patient 176	56	3	95	76	59	95	70	49	91	52	28	88	57	34
Patient 177	25	5	93	69	51	86	34	12	88	41	18	91	31	9
Patient 178	37	11	81	46	28	87	37	14	92	54	30	81	45	16
Patient 179	51	5	93	69	51	94	65	43	95	67	47	95	77	60
Patient 180	96	5	93	69	51	97	83	69	97	81	66	98	78	61
Patient 181	96	9	90	62	43	96	74	55	96	77	60	94	81	64
Patient 182	31	9	90	62	43	93	61	38	94	66	44	88	51	34
Patient 183	61	12	81	46	28	93	58	35	90	46	23	95	75	61
Patient 184	25	7	90	62	43	88	41	17	86	32	11	90	40	20
Patient 185	56	7	90	62	43	97	81	65	98	86	74	94	77	59
Patient 186	32	5	93	69	51	97	80	64	96	74	56	97	58	45
Patient 187	94	7	90	62	43	97	79	63	98	84	72	89	72	57
Patient 188	9	23	53	19	9	96	73	54	93	58	35	65	44	26
Patient 189	28	9	90	62	43	93	62	39	92	56	33	91	62	42
Patient 190	93	9	90	62	43	97	79	64	97	81	67	97	92	79
Patient 191	35	5	93	69	51	97	83	70	98	88	78	96	46	33
Patient 192	92	5	93	69	51	97	79	63	97	80	66	98	87	69
Patient 193	92	3	95	76	59	98	87	76	98	89	80	98	86	81
Patient 194	36	12	81	46	28	94	64	41	94	66	45	95	74	49
Patient 195	41	5	93	69	51	97	84	70	97	82	68	99	86	64
Patient 196	27	5	93	69	51	94	63	40	92	55	31	94	50	21
Patient 197	91	5	93	69	51	96	74	55	93	58	35	95	83	66
Patient 198	91	12	81	46	28	96	75	57	96	75	58	91	74	53
Patient 199	90	5	93	69	51	95	72	52	96	74	56	99	83	68
Patient 200	89	0	97	82	66	96	75	57	98	83	70	99	79	71
Patient 201	8	3	95	76	59	96	76	58	97	79	63	88	63	40
Patient 202	89	2	95	76	59	99	91	82	98	89	79	98	89	84
Patient 203	20	5	93	69	51	90	46	22	89	42	19	96	44	19
Patient 204	89	12	81	46	28	94	64	42	95	67	46	93	81	60
Patient 205	88	5	93	69	51	96	75	57	96	73	55	98	77	65
Patient 206	72	15	75	38	21	93	60	37	91	50	26	87	66	50
Patient 207	34	5	93	69	51	96	73	54	96	75	57	99	61	39
Patient 208	33	5	93	69	51	92	55	31	93	60	37	98	56	36
Patient 209	46	11	81	46	28	93	59	35	91	50	27	88	53	32
Patient 210	88	5	93	69	51	98	88	78	98	84	72	99	93	79
Patient 211	52	16	68	31	17	93	62	39	95	68	48	92	75	39
Patient 212	86	3	95	76	59	98	90	81	98	84	72	99	93	90
Patient 213	86	3	95	76	59	97	79	63	98	87	77	94	78	56
Patient 214	86	12	81	46	28	97	78	62	96	77	60	95	85	70

Patient 215	86	3	95	76	59	99	91	83	98	89	80	99	96	94
Patient 216	58	5	93	69	51	96	76	58	96	72	54	92	78	56
Patient 217	81	5	93	69	51	97	80	64	98	85	73	98	77	53
Patient 218	86	2	95	76	59	96	76	58	97	80	65	95	83	71
Patient 219	25	3	95	76	59	95	71	51	94	64	42	98	37	17
Patient 220	84	3	95	76	59	99	91	82	98	88	79	99	97	95
Patient 221	84	12	81	46	28	94	63	40	94	65	43	94	76	50
Patient 222	84	2	95	76	59	97	83	69	97	81	66	97	79	70
Patient 223	34	14	81	46	28	96	74	55	97	79	63	97	85	64
Patient 224	84	3	95	76	59	97	83	69	96	76	59	98	87	75
Patient 225	25	5	93	69	51	94	65	43	91	51	27	97	63	45
Patient 226	57	5	93	69	51	94	63	40	95	68	48	95	62	29
Patient 227	83	3	95	76	59	98	85	73	98	89	80	99	92	82
Patient 228	33	9	90	62	43	95	71	51	97	79	63	95	81	60
Patient 229	54	5	93	69	51	91	50	26	90	48	24	93	49	20
Patient 230	83	5	93	69	51	97	82	68	98	87	77	98	86	77
Patient 231	21	2	95	76	59	95	72	53	96	74	56	99	56	37
Patient 232	61	5	93	69	51	94	64	42	91	50	26	97	71	60
Patient 233	16	7	90	62	43	90	47	22	89	44	21	95	35	17
Patient 234	81	3	95	76	59	97	80	65	94	65	44	99	85	79
Patient 235	51	10	86	54	35	97	81	67	96	77	60	93	84	55
Patient 236	28	3	95	76	59	97	78	62	94	62	39	96	62	50
Patient 237	81	5	93	69	51	97	82	68	98	87	76	97	81	70
Patient 238	31	5	93	69	51	94	65	42	91	52	28	98	66	54
Patient 239	81	3	95	76	59	98	86	74	97	81	66	95	74	56
Patient 240	12	9	90	62	43	85	32	11	85	32	11	88	35	18
Patient 241	81	3	95	76	59	97	84	70	98	89	79	99	90	81
Patient 242	13	11	81	46	28	89	44	19	91	51	27	93	42	20
Patient 243	80	5	93	69	51	97	79	62	97	78	62	97	87	75
Patient 244	80	5	93	69	51	98	85	73	98	88	78	99	94	83
Patient 245	80	5	93	69	51	96	74	56	92	55	32	94	80	64

BPI = Basingstoke Predictive Index; CoxPH = Cox Proportional Hazard; RSF = Random Survival Forest

Table S5. 1, 3 and 5-Year Survival Predictions for the 105 Internal Validation Set Patients: BPI versus CoxPH versus DeepSurv versus RSF

		BPI Predictions				CoxPH Predictions			DeepSurv Predictions			RSF Predictions		
	Actual survival by months	BPI Score	1-year %	3-year %	5-year %	1-year %	3-year %	5-year %	1-year %	3-year %	5-year %	1-year %	3-year %	5-year %
Patient 1	6	7	90	62	43	92	57	33	93	57	34	93	51	17
Patient 2	79	12	81	46	28	94	66	44	95	71	52	98	70	48
Patient 3	79	5	93	69	51	98	88	78	98	84	72	98	92	80
Patient 4	79	14	81	46	28	96	77	61	96	76	58	93	79	54
Patient 5	36	3	95	76	59	92	54	30	89	42	19	96	30	15
Patient 6	78	3	95	76	59	93	59	35	93	58	35	93	56	24
Patient 7	78	7	90	62	43	97	82	68	96	76	58	99	81	70
Patient 8	62	11	81	46	28	94	65	42	92	53	29	85	60	34
Patient 9	39	6	90	62	43	98	88	77	97	82	69	97	85	77
Patient 10	0	7	90	62	43	97	79	63	97	81	67	98	66	45
Patient 11	76	9	90	62	43	98	89	79	99	91	83	96	92	88
Patient 12	11	14	81	46	28	89	45	21	92	54	30	94	67	44
Patient 13	13	9	90	62	43	89	44	20	91	50	27	95	59	40
Patient 14	74	4	95	76	59	94	63	40	94	63	40	90	50	21
Patient 15	74	7	90	62	43	97	82	67	97	79	63	97	87	76
Patient 16	72	14	81	46	28	89	44	20	92	55	31	93	64	39
Patient 17	40	7	90	62	43	92	56	32	92	53	29	87	54	41
Patient 18	34	9	90	62	43	97	82	68	97	80	65	95	73	53
Patient 19	70	5	93	69	51	97	82	68	97	80	65	98	80	57
Patient 20	18	9	90	62	43	89	46	21	91	52	28	94	59	40
Patient 21	70	3	95	76	59	99	91	83	98	89	80	99	97	95
Patient 22	70	7	90	62	43	96	74	55	97	81	66	96	79	62
Patient 23	39	5	93	69	51	94	66	44	97	78	62	95	75	65
Patient 24	17	14	81	46	28	95	72	52	94	64	42	91	76	52
Patient 25	69	3	95	76	59	99	91	83	98	89	79	99	96	94

Patient 26	68	7	90	62	43	94	67	45	97	79	64	97	52	29
Patient 27	55	3	95	76	59	94	66	44	92	55	31	95	61	43
Patient 28	40	5	93	69	51	96	74	56	98	84	71	99	75	65
Patient 29	67	5	93	69	51	94	66	44	97	80	64	97	39	17
Patient 30	34	3	95	76	59	96	75	57	95	71	51	92	79	60
Patient 31	10	9	90	62	43	97	79	63	97	80	65	90	53	34
Patient 32	65	5	93	69	51	98	87	76	98	84	71	93	69	52
Patient 33	65	3	95	76	59	96	76	59	96	75	57	98	81	66
Patient 34	65	7	90	62	43	95	71	50	96	75	57	97	68	43
Patient 35	64	14	81	46	28	95	69	48	96	77	60	90	74	58
Patient 36	64	12	81	46	28	96	77	60	96	75	58	93	80	54
Patient 37	63	5	93	69	51	94	64	41	92	55	31	94	50	22
Patient 38	38	16	68	31	17	86	35	13	90	45	21	79	46	25
Patient 39	61	5	93	69	51	96	74	56	97	81	67	95	78	61
Patient 40	61	14	81	46	28	93	61	38	95	67	46	93	75	39
Patient 41	60	7	90	62	43	95	71	52	95	69	49	94	69	52
Patient 42	59	5	93	69	51	86	35	13	89	42	19	92	32	10
Patient 43	59	5	93	69	51	94	67	45	90	47	23	97	79	65
Patient 44	45	9	90	62	43	97	83	70	96	76	59	99	84	74
Patient 45	43	7	90	62	43	91	52	28	91	50	26	77	42	24
Patient 46	59	3	95	76	59	96	77	60	96	77	61	99	68	49
Patient 47	8	7	90	62	43	97	79	63	97	80	66	97	84	66
Patient 48	12	5	93	69	51	95	69	48	94	64	42	92	65	49
Patient 49	29	5	93	69	51	93	58	35	92	56	32	92	67	53
Patient 50	11	9	90	62	43	96	73	54	97	78	61	99	86	71
Patient 51	50	3	95	76	59	96	75	57	97	78	62	89	61	38
Patient 52	22	10	86	54	35	94	65	43	96	73	54	96	74	54
Patient 53	15	7	90	62	43	96	73	54	97	80	66	96	80	61
Patient 54	55	5	93	69	51	89	45	21	90	48	24	97	58	36
Patient 55	31	12	81	46	28	90	49	25	91	49	25	94	58	34
Patient 56	16	4	95	76	59	96	73	55	97	82	68	99	76	65
Patient 57	53	9	90	62	43	95	70	50	95	69	48	96	66	47
Patient 58	52	7	90	62	43	94	66	44	92	56	33	95	72	56
Patient 59	51	14	81	46	28	96	77	60	96	75	58	94	81	55
Patient 60	49	5	93	69	51	98	90	81	98	86	75	99	93	90
Patient 61	49	3	95	76	59	95	72	53	93	58	35	94	57	42
Patient 62	49	9	90	62	43	98	87	76	98	84	71	96	74	60
Patient 63	47	7	90	62	43	95	69	48	97	77	61	99	75	58
Patient 64	47	3	95	76	59	97	80	65	95	67	46	99	82	74
Patient 65	11	7	90	62	43	95	70	50	95	70	50	96	54	35
Patient 66	7	5	93	69	51	92	57	33	89	42	18	95	52	37
Patient 67	46	5	93	69	51	98	86	74	97	82	68	99	86	81
Patient 68	46	2	95	76	59	95	72	52	93	61	39	98	77	65
Patient 69	46	9	90	62	43	98	89	80	98	85	74	99	90	79
Patient 70	45	7	90	62	43	95	68	47	94	64	42	92	63	47

Patient 71	43	3	95	76	59	93	59	35	92	57	34	97	59	33
Patient 72	43	3	95	76	59	97	80	64	98	89	81	98	76	67
Patient 73	17	5	93	69	51	94	66	44	97	79	63	97	59	31
Patient 74	42	7	90	62	43	98	87	75	97	83	70	93	61	43
Patient 75	42	5	93	69	51	91	53	28	91	50	26	95	54	36
Patient 76	12	7	90	62	43	94	64	41	95	68	48	98	70	43
Patient 77	5	3	95	76	59	89	46	21	91	49	25	97	58	36
Patient 78	41	5	93	69	51	93	60	37	93	58	34	98	72	61
Patient 79	41	5	93	69	51	94	64	41	96	76	59	95	72	58
Patient 80	18	5	93	69	51	97	80	65	98	85	74	98	79	60
Patient 81	17	5	93	69	51	94	66	44	97	79	63	96	69	57
Patient 82	19	14	81	46	28	96	77	59	96	73	54	93	80	51
Patient 83	49	7	90	62	43	98	89	80	98	85	74	99	93	90
Patient 84	59	5	93	69	51	92	56	32	89	42	19	95	52	36
Patient 85	98	5	93	69	51	96	78	61	97	80	65	95	69	43
Patient 86	30	5	93	69	51	97	83	70	97	82	68	98	82	74
Patient 87	94	3	95	76	59	98	87	77	98	84	71	99	90	77
Patient 88	93	3	95	76	59	97	82	68	97	82	68	98	86	68
Patient 89	82	5	93	69	51	98	84	71	97	82	68	99	91	77
Patient 90	19	7	90	62	43	94	63	40	96	75	57	95	51	29
Patient 91	70	5	93	69	51	96	77	60	96	77	60	99	85	70
Patient 92	90	7	90	62	43	95	68	47	97	79	63	99	50	29
Patient 93	89	5	93	69	51	96	76	59	97	81	67	95	73	58
Patient 94	89	5	93	69	51	97	81	67	97	81	66	99	87	73
Patient 95	88	5	93	69	51	97	82	68	98	87	76	98	80	68
Patient 96	88	3	95	76	59	93	59	35	92	53	29	88	51	23
Patient 97	33	14	81	46	28	93	60	36	93	59	36	93	59	35
Patient 98	24	7	90	62	43	95	70	49	95	68	47	89	61	35
Patient 99	16	12	81	46	28	88	42	18	92	54	30	97	58	32
Patient 100	22	12	81	46	28	87	39	15	89	44	21	93	54	35
Patient 101	51	3	95	76	59	95	70	50	96	72	53	99	72	50
Patient 102	83	3	95	76	59	98	86	74	97	82	69	99	67	55
Patient 103	82	2	95	76	59	95	71	51	96	74	55	99	82	63
Patient 104	35	5	93	69	51	95	72	52	96	75	57	96	75	62
Patient 105	69	7	90	62	43	97	82	68	98	87	77	98	86	77

BPI = Basingstoke Predictive Index; CoxPH = Cox Proportional Hazard; RSF = Random Survival Forest

Table S6. SHAP Explainer Settings Across Survival Models

Model	Explainer	Output	Background Set

CoxPH	Kernel	Risk Score	Full training cohort (245)
RSF	Kernel	Risk Score	245-center k-means summary
XGBoost	Kernel	Risk Score	245-center k-means summary
SVM	Kernel	Risk Score	245-center k-means summary
DeepSurv	Deep	Risk Score	Full training cohort (245)
DeepHit	Deep	Risk Score	Full training cohort (245)

Table S7. Hyperparameter Search Space

Model	Parameter	Search Space
CoxPH	alpha (L2 / ridge penalty)	log-uniform [1e-8, 1e2]
	ties-handling method	{breslow, efron}
	CV folds	{3, 4, 5}
	Regularization	L2 (ridge) penalty via alpha (tuned)
	Seed	42
RSF	n_estimators	int [25, 500]
	max_depth	int [1, 200]
	min_samples_split	int [2, 50]
	min_samples_leaf	int [1, 25]
	CV folds	{3, 4, 5}
	Regularization	Tree-depth / leaf-size constraints (tuned) + ensemble averaging across n_estimators trees

	Seed	42
XGBoost	n_estimators	int [50, 500]
	learning_rate	log-uniform [1e-5, 0.3]
	max_depth	int [2, 8]
	validation_fraction	[0.05, 0.3]
	n_iter_no_change (early stopping)	int [5, 20]
	tol (early-stopping tolerance)	log-uniform [1e-5, 1e-2]
	CV folds	{3, 4, 5}
	Regularization	Shrinkage via learning_rate + max_depth constraint + early stopping (n_iter_no_change / tol / validation_fraction), all tuned
	Seed	42
SVM	alpha (regularization)	log-uniform [1e-3, 10.0]
	kernel	{linear, rbf, poly}
	gamma (rbf/poly only)	log-uniform [1e-4, 1.0]
	CV folds	{3, 4, 5}
	Regularization	L2-type penalty via alpha (tuned)
	Seed	42
DeepSurv	architecture	(64,64) / (64,64,64) / (32,32) / (32,32,32) / (16,16) / (16,16,16); fully connected, batch norm
	dropout	{0.10, 0.15, 0.20, 0.25, 0.30, 0.35}
	batch size	{16, 32, 64}
	learning rate	log-uniform [1e-4, 1e-2]
	weight decay (L2, Adam)	log-uniform [1e-6, 1e-2]
	CV folds	{3, 4, 5}

	output layer	1 unit (Cox log-relative-hazard g(x))
	Optimizer / early stopping	Adam; early stopping on validation loss, patience = 20 epochs, max 300 epochs
	Regularization	Dropout (tuned) + Adam weight decay (tuned, L2)
	Seed	42
DeepHit	time discretization (num_durations)	int [3, 15], equidistant binning over observed follow-up
	architecture	same 6 choices as DeepSurv
	dropout	{0.10, 0.15, 0.20, 0.25, 0.30, 0.35}
	batch size	{16, 32, 64}
	alpha (loss-mixing weight)	[0.05, 0.35]
	sigma (ranking-loss smoothing)	[0.05, 0.35]
	CV folds	{3, 4, 5}
	output layer	num_durations units (one per discretized time bin)
	Optimizer / early stopping	Adam; early stopping via torchtuples default patience = 10 epochs (not overridden in code), max 300 epochs
	Regularization	Dropout (tuned) + ranking-loss smoothing sigma (tuned); weight decay not applied to optimizer
	Seed	42

CoxPH = Cox Proportional Hazard; RSF = Random Survival Forest; SVM = Support Vector Machine
